# Frequency of alpha-1 antitrypsin deficiency and unexpected results in COPD patients in Turkey; rare variants are common

**DOI:** 10.55730/1300-0144.5486

**Published:** 2022-08-30

**Authors:** Mustafa ÇÖRTÜK, Barış DEMİRKOL, Melih Akay ARSLAN, Umut İLHAN, Yunus Emre KALKAN, Demet TURAN, Şule GÜL, Halit ÇINARKA, Kürşad Nuri BAYDİLİ, Erdoğan ÇETİNKAYA

**Affiliations:** 1 Department of Pulmonology, Yedikule Chest Diseases and Thoracic Surgery Education and Research Hospital, University of Health Sciences, İstanbul, Turkey; 2 Department of Pulmonology, Başakşehir Cam and Sakura City Hospital, University of Health Sciences, İstanbul, Turkey; 3 Department of Biostatistics and Medical Informatics, Faculty of Medicine, University of Health Sciences, İstanbul, Turkey

**Keywords:** Alpha 1-antitrypsin deficiency, chronic obstructive pulmonary disease, screening, mutation, genome, gene frequency

## Abstract

**Background/aim:**

Alpha-1 antitrypsin (α1-AT) is a protease inhibitor that is largely released from liver cells. It inhibits neutrophil elastase and its deficiency increases the risk of developing chronic obstructive pulmonary disease (COPD). The frequency of α1-AT deficiency has been reported with different prevalence rates in different parts of the world. The most common α1-AT variant causing α1-AT deficiency is the Pi*Z allele. In this study, we aimed to determine the frequency of the α1-AT genotypic variant in COPD patients in our country.

**Materials and methods:**

In this study, 196 consecutive COPD patients admitted to our clinic were included. In addition to recording the demographic data of the volunteers, a dry drop of blood sample was taken from the fingertip for the SERPINA1 genotype study.

**Results:**

One hundred and fifty-eight (80.6%) of the patients were male and the mean age was 56.92 ± 9.84 years. A variant in the SERPINA1 gene was detected in a total of 14 (7.1%) COPD patients. Pi*ZZ homozygous variant was detected in only 1 (0.51%) patient, while Pi*MZ was detected in 3 (1.53%) patients. The Pi*S variant was never detected. Various rare heterozygous variants were detected in 9 (4.6%) patients and a single point mutation was found in one (0.51%) patient. Serum α1-AT levels were significantly lower in patients with variants compared to the Pi*MM group (p < 0.001).

**Conclusion:**

In this study, which investigated the genotypic α1-AT variant frequency in COPD patients for the first time in our country, we found that the percentage of homozygous Pi*ZZ patients was 0.51%, but when heterozygous α1-AT gene variant and single point mutation were included, the frequency was 7.1%. At the same time, while the Pi*S variant was never detected, rare variants were found more frequently than expected.

## 1. Introduction

Alpha-1 antitrypsin (α1-AT) is a single-chain glycoprotein consisting of 394 amino acids and 52kDa in size [1]. The main function of α1-AT encoded by the SERPINA1 gene is to prevent excessive destruction of elastin and collagen tissue by inactivating elastase and proteinase 3 released from activated neutrophils [2]. Pulmonary pathologies that occur when the serum level of α1-AT, which shows this activity most intensely in the liver and lung, is insufficient, is a characteristic feature of alpha-1 antitrypsin deficiency (AATD). Although emphysema and chronic obstructive pulmonary disease (COPD) are the most common conditions that occur in the lung, bronchiectasis of varying severity may also be seen in some patients [3].

M-type protease inhibitor (Pi) is the most common wild-type allele of α1-AT in the general population. Pi*MM individuals have “normal” plasma levels of α1-AT (>20 μmol/L), which is sufficient to bind and inactivate neutrophil elastase. It has been reported that the common variation among Pi variants in patients with AATD is Z and S variants. Those with homozygous genes for the Z allele are expressed as Pi*ZZ and have plasma α1-AT levels around 5–6μmol/L, while plasma levels of Pi*SS individuals have approximately 60% of normal (14–20μmol/L) [1,4]. The risk for lung disease due to alpha-1 antitrypsin deficiency depends on the individual’s α1-AT genotype and α1-AT serum levels as well as environmental factors. Data on the epidemiology of remaining missing α1-AT variants, some of which are associated with significant reduction or absence of plasma α1-AT, are limited. Because of their low frequency, these non-Pi*S and non-Pi*Z missing variants are termed ‘rare’ [5].

It has been reported that there is a variable frequency of AATD in studies conducted in different regions of the world. For example, while the frequency is 1/5771 in the central region of Europe, it is reported as 1/35702 in the east and 1/3785 in the south [6]. It has been estimated to be 1/4126 among non-Hispanic whites in the United States [6]. It is important to know the regional frequency of AATDs and detect them because AATD is the most well-known familial cause of COPD and individualized treatment with α1-AT replacement can be applied.

There are data on the frequency of AATD, especially in developed countries of the world. Although there are few AATD-related studies conducted with the general population and other patient groups in our country, as far as we know, there is no study in which the frequency of AATD was determined in COPD patients. For this reason, a SERPINA1 genotype study was conducted on consecutive COPD patients admitted to our hospital to determine the frequency of AATD in COPD patients in our country.

## 2. Materials and methods

This prospective cross-sectional study was conducted between February 2020 and February 2022 after local ethics committee approval.

All COPD patients who applied to our clinic in our center, which is a 3rd level branch hospital of Chest Diseases and Thoracic Surgery, were evaluated. After the written consent of the patients was obtained, their demographic characteristics and comorbidities were recorded. A dry drop blood sample was taken for the genotype study. In addition, serum α1-AT levels, alanine transaminase (ALT), aspartate transaminase (AST) levels, and 6-min walking distance (6-MWT) were recorded. The patients were recorded as emphysema-predominant, chronic bronchitis-predominant, and mixed-type COPD based on their clinical and radiological features.

We used serum samples and dried blood spot (DBS) samples (AlphaKits® GE Healthcare Ltd, Cardiff, CF147YT, UK) for screening and testing for AATD. The genotype study was conducted at the Progenika Biopharma laboratory in Spain. Pulmonary function tests (PFTs) were performed by using the Sensormedics model 2400 (Yorba Linda, CA, USA) spirometry device, and according to the American Thoracic Society (ATS) guidelines [7].

### 2.1. Statistical analysis

Analysis of the data was carried out using the SPSS 25 package program. Frequency and percentage values are presented for qualitative variables. The conformity of the data to the normal distribution was tested with the Shapiro-Wilk test. While the arithmetic means and standard deviation values are presented for the variables conforming to the normal distribution, the median, minimum, and maximum values are presented for the variables not conforming to the normal distribution. In comparisons between two-category qualitative variables and quantitative variables; an independent sample t-test was used if parametric test assumptions were met, Mann-Whitney U test was used if parametric test assumptions were not met. Type I error rate was taken as 0.05 in the study.

## 3. Results

A total of 196 COPD patients, of which 158 (80.6%) were male, were included in the study consecutively. The mean age of the volunteers was 56.92 ± 9.84 years. Considering their smoking history, 39 patients (19.9%) had never smoked. In patients who were active smokers (25.5%) or quit smoking (54.6%), the mean duration of smoking was 35 (min-max 1–120) pack years. Ninety-three (47.4%) patients had comorbid diseases. Demographic characteristics, comorbidities, smoking history, pulmonary function tests, ALT, AST, 6-MWT results of the patients are given in Table 1.

A variant in the SERPINA1 gene was detected in 14 (7.1%) of the patients. While Pi*ZZ homozygous mutation was detected in only 1 (0.51%) patient, various heterozygous anomalies were detected in 12 (6.12%) patients. A single-point mutation was detected in one patient (Table 2). In the comparison made between COPD patients with and without a variant in the SERPINA1 gene, there was a statistical difference between serum α1-AT levels, but no difference was found in other parameters (Table 3).

However, while the mean serum α1-AT level was 146 mg/dL in Pi*MM patients, it was 100 mg/dL when all variants were included, and 119 mg/dL when Pi*MZ and Pi*ZZ were excluded, which was statistically significant (respectively p =< 0.001 and p = 0.025). In addition, it was determined that there was no difference between COPD patients with and without variants in the SERPINA1 gene in terms of emphysema-predominant, chronic bronchitis-predominant, and mixed COPD (0.462). The HRCT section of the patient with a homozygous ZZ variant is presented in the figure.

## 4. Discussion

COPD is a chronic respiratory disease that has risen to the top of the list of diseases that cause mortality in recent years. Well-known environmental risk factors for COPD include air pollution, particulate matter, and prolonged exposure to irritating gases, particularly cigarette smoke. In addition to environmental factors, genetic factors also play a role in the development of COPD, and AATD is the best known genetic factor [1]. Therefore, the World Health Organization reported that all individuals with COPD should be evaluated for AATD, regardless of smoking history and phenotype [8].

Alpha-1 antitrypsin is a major lung serine proteinase inhibitor and protects the lung parenchyma from neutrophil-associated proteolytic degradation. In case of insufficient serum level or function, COPD is thought to occur with the contribution of other environmental factors. The first cases of AATD deficiency were reported in 1963 by Laurell and Eriksson in five individuals, three of whom had significant emphysematous lung disease [9]. In other studies, conducted later, it was found that COPD generally has an earlier onset in α1-AT deficiency and panlobular emphysema, which is dominant at baseline, develops [2]. Therefore, the COPD pattern with predominant baseline and prominent panlobular emphysema has been accepted as the classical clinical phenotype. In previous years, those with more classical phenotypes and severe lung disease were investigated for α1-AT. However, current guidelines suggest that all COPD patients should be checked for α1-AT levels, regardless of age, smoking status, and COPD severity, since current information shows that AATD can also occur in those who do not have the classical phenotype. In our cases who were homozygous and heterozygous for α1-AT deficiency, there was no clinically significant difference from other COPD cases, except that the patient with homozygous Pi*ZZ was relatively younger. In addition, no statistically significant difference was found in terms of emphysema or chronic bronchitis-predominant COPD between our patients with and without variants, in line with the current recommendations. However, our patient with Pi*ZZ homozygous was emphysema dominant type.

The frequency of AATD has a different prevalence in various countries of the world. Previously, Blanco et al. in the study, in which 224 cohorts from 65 countries were evaluated, Pi*Z and Pi*ZZ were the highest frequency and prevalence in the Atlantic and coastal regions of Europe, while it was decreasing gradually in Eastern Europe, and Asia, it was moderate in Pakistan and Thailand. It has been shown to nearly disappear in Asian regions. Moderate Pi*Z values were detected in some parts of East Africa, while very low or no Pi*Z values were found in the rest of Africa [6]. In our country, there is only one study in which the serum α1-AT level of COPD patients was studied before [10]. Apart from this, there are a limited number of studies conducted on patients with interstitial lung diseases (ILD) and in the pediatric age group [11,12]. To our knowledge, our study is the first study in our country with genotypical AATD screening results in unselected COPD patients admitted consecutively.

In the study conducted by Simsek et al., using serum samples collected from 1203 healthy individuals in our country, the allele frequencies by isoelectric focusing method were 96.8% for Pi*MM, 0.7% for Pi*MZ, 0.6% for Pi*MS, 0.5% for Pi*MF and Pi. *M? (unidentified variants with available standards) were reported as 1.4% [13]. As a result of this study, it was calculated that 7256 people in our country should have Pi*ZZ disease. The only study we could find in Turkey regarding α1-AT deficiency in COPD patients was the study by Perincek et al. in which serum α1-AT levels were screened in COPD patients [10]. In this study, serum α1-AT level was measured in COPD patients without genotypic analysis, and its relationship with the COPD stage was examined. As a result, it was determined that the serum α1-AT level was below the reference value in only one of the 243 patients admitted to the hospital, and the serum α1-AT level increased in direct proportion to the stage of COPD. In our study, the variant was detected in 14 (7.1%) of the patients who applied to our clinic consecutively, in the gene analysis performed. Among these variants, only one patient had a Pi*ZZ homozygous mutation, Pi*MZ, Pi*MMalton, and Pi*MI variants were detected in three patients, each one with Pi*MProcidia, a total of 12 patients with various heterozygous anomalies, and one patient with a single nucleotide polymorphism was detected. The patient with Pi*ZZ was 44 years old and α1-AT replacement therapy was started.

The normal allele for Alpha 1 antitrypsin is Pi*MM. So far, more than 120 SERPINA1 mutations have been reported in the literature, and about 40% of them have been reported to cause AATD [14]. The most common deficiency alleles are Pi*S and Pi*Z, which encode abnormal proteins that polymerize in the liver. The normal genotype, Pi*MM, is found in approximately 80%–95% of the population, while almost all of the rest of the population consists of variants (Pi*SS, Pi*MZ, Pi*SZ, and Pi*ZZ) that are a combination of the M, Z, and S alleles [1]. The epidemiology of other variants, some of which are associated with significant reduction or absence of plasma α1-AT, is poorly known and due to their low frequency, these non-S and non-Z variants are termed “rare” [5]. There are approximately 25 rare deficiency alleles associated with reduced serum α1-AT levels and 25 null alleles associated with undetectable amounts of serum α1-AT levels [15]. While the prevalence of missing genotypes other than common PI*ZZ or PI*SZ among individuals with AATD in Italy was 11%, the prevalence of missing genotypes other than PI*MZ was reported to be 15% in individuals with moderate AATD [16]. In Portugal, among those with AATD deficiency, a remarkable ratio of hetero- or homozygous rare alleles and null alleles were detected in 9.5% of cases [17]. In a recently published review, it was reported that a large number of mutations could be detected, and rare mutations could be detected in up to 17% of clinical cases in studies, but they were usually detected at much lower frequencies [18]. In this study, the Pi*S allele, one of the most common variants, was not detected in any of the patients. However, among all variants detected, heterozygous Pi*MZ, Pi* MMalton, and Pi* MI alleles were equally numerous, with three each (21.4%). In addition, Pi*MPlowel was detected in 2 (14.2%) patients and Pi*Mprocida in 1 (7.1%) patient. Alleles named “rare” in our study were found to be more common than previously reported rates in other studies. The reason why the Pi*S variant was never detected and the expected number of rare variants could not be explained. Although the available data are not sufficient to generalize the frequency of rare variants in our country, our present findings clearly reveal the need for more extensive studies in this area.

During our study, full gene sequencing was requested in a 40-year-old female patient with COPD accompanying bronchiectasis and previous pulmonary embolism, because of GOLD stage IV. Sequencing of SERPINA1 (NM_001127701.1) full gene was carried out and the following variant is detected: Status Heterozygous, Genome position (GRCh38) g.94378611 was detected. However, serum α1-AT level was measured three times during stable periods and was found to be normal. In previously published studies, no direct literature information indicating AATD deficiency with this mutation could be reached.

The precise determination of the risk of developing COPD in those with heterozygous Pi*MZ and the contribution of environmental effects such as smoking continue to be investigated. The consensus is that smoking increases the risk for impaired lung function and the development of COPD in those with the heterozygous variant. A longitudinal study showed that individuals with the Pi*MZ genotype had a slightly greater annual reduction of FEV1 (25 vs. 21 mL/year, p = 0.048) compared with those with the Pi*MM genotype. In addition, the odds ratio was found to be 1.3 for the development of airflow obstruction in Pi*MZ heterozygotes compared to normal Pi*MM homozygotes [19]. All of the heterozygous patients in our study had a history of smoking. For this reason, clear data could not be presented on whether smoking or not smoking contributes to the development of COPD.

In the presence of Pi*Z, the most common and most important variant, a low serum α1-AT level is the expected result. However, in the presence of rare variants, the exact results of serum α1-AT levels are not known. In a study conducted in Italy, rare mutations were found in 22 patients in the screening of 200 patients with respiratory pathology, and serum α1-AT levels were found to be low in these patients [20]. Similarly, in our study, there was a statistically significant difference in serum α1-AT levels between those with Pi*MM and both all variants and other rare variants (except Pi*ZZ and Pi*MZ). However, a statistical comparison between rare variants could not be made due to the insufficient number of rare variants. Therefore, an off-label application was made in 2 of our heterozygous patients due to low serum α1-AT levels and replacement therapy was started.

In clinical practice, more than 95% of severe AATD cases are associated with the Pi*ZZ genotype, some of them may occur with liver-related diseases in childhood, COPD may develop at later ages, especially in the case of cigarette exposure, and less frequently neutrophilic panniculitis, polyangiitis granulomatosis, and hepatocarcinoma have been reported [21]. Accumulation of α1-AT protein in hepatocytes can be seen in some variants, most commonly Pi*Z and Pi*MMalton alleles, in which case there is an increased risk of liver disorders such as neonatal hepatitis, cirrhosis that may occur in childhood or adulthood, and hepatocellular carcinoma [22]. In our study, liver enzymes of alanine transaminase and aspartate transaminase levels were recorded in patients who applied for this reason, but no difference in liver enzymes was found between patients with homozygous or heterozygous variants and those with normal variants. In the patient with homozygous Pi*ZZ, additional abdominal ultrasonography was performed and no significant abnormality was detected. Volunteers were also compared in terms of functional parameters, but no difference was found between pulmonary function tests and 6-min walk tests between patients with homozygous and heterozygous variants and those with normal variants. However, as expected, serum α1-AT levels were found to be lower in patients with variants.

One of the limitations of this study is that the number of patients is not at a level to reflect the whole country. In addition, since it is single-centered, it will not be able to show possible differences between regions.

In conclusion, in this study, which investigated the genotypic AATD frequency in COPD patients for the first time in our country, we found that the frequency of homozygous and heterozygous AAT gene variants was higher than we thought. Also, while the Pi*S variant was never detected, rare variants were found more frequently than expected. This study shows us that AATD deficiency should always be kept in mind and investigated in COPD patients and that similar studies should be conducted on a larger scale in our country.

## Figures and Tables

**Figure:**
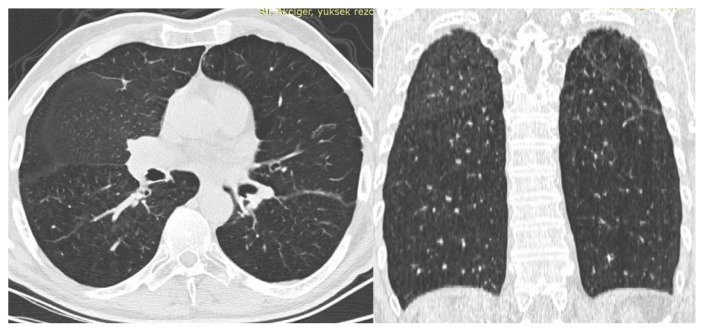
In the axial and coronal sections of the tomography of a patient with alpha 1 antitrypsin deficiency, more prominent diffuse emphysema is seen in the lower lobes.

**Table 1:** Demographic, basic laboratory, and functional parameters of the patients.

	f (%)
**Gender**	
Male	158 (80.6)
Female	38 (19.4)
**Comorbidity**	
No	103 (52.6)
Yes	93 (47.4)
DM	24 (12.2)
HT	31 (15.8)
ILD	4 (2)
CHF	8 (4.1)
CVD	1 (0.5)
Sleep apnea syndrome	1 (0.5)
Ischemic heart disease	22 (11.2)
Others	40 (20.4)
Smoking status	
Nonsmoker	39 (19.9)
Smoker	50 (25.5)
Ex-smoker	107 (54.6)
Package/year med (min- max)	35 (1–120)
	*x′* ± *SS***/Med (min-max)**
Age	56.92 ± 9.84
Serum α1-AT level	144 (32–237)
AST	19 (7–74)
ALT	18 (4–95)
FEV1	1.64 (0.33–4.81)
%FEV1	55 (12–130)
FVC	2.51 (0.84–6.44)
%FVC	71.34 ± 22.22
FEV1/FVC	0.65 ± 0.19
TLC	6.85 (3.2–11.2)
%TLC	38.55 ± 57.81
RV	4.4 (0.04–9.4)
%RV	97.22 ± 130.85
6-MWT	365 (2.3–700)

DM: Diabetes mellitus, HT: Hypertension, ILD: Interstitial lung disease CHF: Congestive heart failure, CVD: Cerebrovascular disease, AST: Aspartate aminotransferase, ALT: Alanine aminotransferase, FEV1: Forced expiratory volume in 1 s, FVC: Forced vital capacity, TLC: Total lung capacity, RV: Residual volume, 6- MWT: 6-min walk test

**Table 2:** SERPINA1 genotypic distribution of volunteers.

Genotype	n (%)
Pi*MM	182 (92.9 %)
Pi*MMalton	3 (1.53 %)
Pi*MZ	3 (1.53%)
Pi*Mprocida	1 (0.51%)
Pi*MI	3 (1.53%)
Pi*MPlowel	2 (1.02%)
Pi*ZZ	1 (0.51%)
Single point mutation (GRCh38) g.94378611	1 (0.51%)

**Table 3:** Comparison of laboratory and functional parameters between those with and without variants in the SERPINA1 gene.

	Variant (−)	Variant (+)	U / t	p
**Serum ** **α** **1-AT level**	146 (32–237)	100 (37.6–164)	298^U^	<0.001*
**AST**	21.05 ± 9.76	20.09 ± 6.44	0.32^t^	0.749
**ALT**	20.63 ± 12.06	23.18 ± 10.53	−0.684 ^t^	0.495
**FEV1**	1.74 ± 0.88	2.11 ± 1.23	−1.39 ^t^	0.166
**%FEV1**	55.88 ± 24.43	67.28 ± 37.64	−1.072 ^t^	0.303
**FEV1/FVC**	0.65 (0.23–1.76)	0.64 (0.33–1.24)	769.5 ^U^	0.289
**FVC**	2.68 ± 1.03	2.92 ± 0.98	−0.817 ^t^	0.415
**FVC%**	70.69 ± 21.38	78.58 ± 30.2	−0.921 ^t^	0.373
**TLC**	6.44 ± 1.91	8.35 ± 4.04	−1.237 ^t^	0.231
**%TLC**	1.23 (0.55–158)	82.42 (0.84–164)	16 ^U^	0.719
**RV**	4.53 ± 1.95	6.42 ± 4.21	−1.194 ^t^	0.247
**%RV**	2.8 (0.96–355)	261 (98–424)	7 ^U^	0.151
**6-MWT**	366.28 ± 194.89	253.25 ± 213.02	1.05 ^t^	0.305
